# Oxycodone relieves permeability damage and apoptosis of oxygen-glucose deprivation/reoxygenation-induced brain microvascular endothelial cells through ras homolog family member A (RhoA)/ Rho-associated coiled-coil containing kinases (ROCK)/ myosin light chain 2 (MLC2) signal

**DOI:** 10.1080/21655979.2022.2037371

**Published:** 2022-02-16

**Authors:** Fang Shao, Dong Han, Yihui Shen, Wen Bian, Liting Zou, Yiqian Hu, Wei Sun

**Affiliations:** Department of Emergency, Affiliated Hospital of Jiangnan University, Wuxi, Jiangsu, China

**Keywords:** Oxycodone, oxygen-glucose deprivation/reoxygenation, permeability damage, RhoA/ROCK/MLC2 signal

## Abstract

Cerebrovascular disease, an important cause of acute ischemic stroke, has attracted worldwide attention. Oxycodone has been widely used to treat various painful disorders. This study was designed to explore the mechanism of oxycodone in oxygen-glucose deprivation/reoxygenation (OGD/R)-induced brain microvascular endothelial cell model. For the reliability of the results in the following experiments, the viability was firstly detected using CCK-8. With the application of LDH, TEER and TUNEL assays, the LDH expression, permeability and apoptosis of brain microvascular endothelial cells were detected, respectively. Besides, the mRNA and protein expressions of tight junction proteins and RhoA were measured using RT-qPCR and Western blot. Moreover, RT-qPCR was employed to evaluate the expressions of inflammatory cytokines. Western blot was adopted to measure the levels of RhoA, ROCK, MLC2 and apoptosis-related proteins. The results revealed that oxycodone attenuated permeability damage, inflammatory factor release and apoptosis of OGD/R-induced brain microvascular endothelial cells in a dose-dependent manner. It was also found that oxycodone could reduce the expressions of RhoA, ROCK and MLC2 in brain microvascular endothelial cells induced by OGD/R. More importantly, oxycodone exhibited desirable effects on OGD/R-induced brain microvascular endothelial cells through RhoA/ROCK/MLC2 signal. In conclusion, oxycodone relieved permeability damage and apoptosis of OGD/R-induced brain microvascular endothelial cells through RhoA/ROCK/MLC2 signal, suggesting that oxycodone might be an effective method for the improvement of cerebral ischemia-reperfusion injury.

## Introduction

Cerebrovascular disease, an extremely complicated pathological process, features high rates of disability and mortality [1]. Previous studies have reported that ischemia-reperfusion injury results from blood reperfusion injury in ischemic brain tissue and usually takes place during the treatment stage of ischemic disease [2–4], thus aggravating the brain tissue injury. Besides, ischemia-reperfusion injury is the contributor to death or different degrees of disability [5]. The pathophysiology of ischemia-reperfusion injury could be divided into ischemic phase and reperfusion phase. In the former phase, the metabolic demand and supply was imbalanced in the ischemic region and in the latter phase, the inflammatory response was activated [6]. Up to now, few effective protocols are available to address ischemia-reperfusion injury and the current therapies showed limited efficacy in alleviating inflammation and neuronal cell apoptosis [7–10]. Notably, ischemia enhances the permeability of brain microvascular endothelial cells, and alterations in the blood-brain barrier are closely associated with oxygen-glucose deprivation/reoxygenation (OGD/R) injury, which could lead to irreversible damage such as endothelial cell apoptosis or death [11]. Therefore, cellular models from OGD/R injury have a very important role in research.

Oxycodone (6-deoxy-7,8-dehydro-14-hydroxy-3-O-methyl-6oxomorphine), μ and κ opioid receptor agonist, has been put into use since 1917 [12]. And oxycodone has been widely used to treat various painful disorders [13]. Since the 1960s in Finland, oxycodone has been commonly used as analgesic for the treatment of postoperative and other acute pain in adults [14]. Elsewhere, oxycodone was testified to alleviate inflammatory injury in brain and ischemia/hypoxia reperfusion-induced neuron injury [15,16]. Moreover, researchers held the opinion that oxycodone could reduce the permeability damage of pulmonary microvascular endothelial cells during lung injury [17]. Although oxycodone exhibited favorable effects on some disease, its mechanism of action on OGD/R-induced brain microvascular endothelial cells remained unexplored.

Ras homolog family member A (RhoA), a ubiquitously expressed cytoplasmic protein, is a member of small GTPases which act as important players in cytoskeletal rearrangement, reactive oxygen species (ROS) production as well as cell morphology [18,19]. It was testified that oxycodone exhibited suppressive effects on myocardial cell apoptosis after myocardial ischemia-reperfusion injury via blocking the RhoA/Rho-associated coiled-coil containing kinase 1 (ROCK1) signaling pathway [20]. In addition, as a RhoA/ROCK downstream signaling factor, activation of myosin light chain 2 (MLC2) was produced in the form of phosphorylation, and papers have demonstrated the involvement of RhoA/ROCK/MLC2 in cerebral ischemia-reperfusion injury to the blood-brain barrier [21–23].

In the present study, oxycodone was investigated for the first time to reduce OGD/R-induced permeability damage and apoptosis in brain microvascular endothelial cells by regulating RhoA/ROCK/MLC2 signaling.

## Material and methods

### Cell culture, treatment and transfection

Mouse brain microvascular endothelial cells (bEND3 cells) that provided by American Type Culture Collection, USA) were incubated in Dulbecco’s modified Eagle’s medium (DMEM) which was supplemented with 10% fetal bovine serum (FBS), 100 U/ml penicillin and 100 μg/ml streptomycin at 37°C in humid atmosphere with 5% CO_2_. bEND3 cells were cultured under OGD/R conditions in order to establish the brain microvascular endothelial cell injury model. At the beginning, bEND3 cells were incubated in glucose-free medium at 37°C in the hypoxic atmosphere (1% O_2_, 5% CO_2_, 94% N_2_) for 5 h, after which the normal medium was utilized to further culture these cells for 24 h at 37°C with 5% CO_2_. Additionally, oxycodone with different concentrations (4 μg/ml, 8 μg/ml, 16 μg/ml) was adopted to treat bEND3 cells.

RhoA overexpression plasmids (Ov-RhoA) and its corresponding negative control (Ov-NC) were obtained from Shanghai Gene Pharma company. The cell transfection was conducted by Lipofectamine™ 2000 transfection reagent (Thermo Fisher Scientific, Inc.).

### Cell Counting Kit-8 (CCK-8)

At the beginning, bEND3 cells were cultured under OGD/R conditions for 5 h, after which CCK-8 was performed. Subsequently, the cells were inoculated into 96-well plates and CCK-8 reagent was added to incubate cells at 37°C for 3 h. Finally, the absorbance at 450 nm was determined by using spectrophotometer (Thermo Fisher Scientific).

### Lactate dehydrogenase (LDH) assay

The loss of membrane plasma integrity was demonstrated by the release of LDH. On a 96-well measuring plate, 100 μl supernatant was added to 100 μl cytotoxicity detection reagent. The activity was determined by colorimetric measurement of sodium pyruvate reduction in the presence of oxycodone [24].

### Transendothelial electrical resistance (TEER)

After establishing the OGD/R-induced injury model, a millicell-ERS apparatus (Millipore) was adopted to measure transendothelial electrical resistance. In order to ensure temperature equilibration and uniformity of culture environment, TEER was recorded 30 min after the medium exchange in each measurement. The background resistance was corrected by collagen coated Transwell inserts which had no cells. The final resistance was calculated by subtracting background resistance from measured barrier resistance, and then multiplying by the effective surface area of the filter membrane.

### Reverse transcription-quantitative PCR (RT-qPCR)

Total RNA from sample cells was lysed with Trlzol® reagent (Thermo Fisher Scientific, Inc.) and then reversely transcribed into complementary DNA (cDNA). PCR for gene quantitation assay using SYBR-Green Supermix (Invitrogen) was carried out on ABI 7500 quantitative PCR instrument (ABI/Perkin Elmer) strictly in line with the manufacture’s protocol. 2^−ΔΔCt^ method was adopted to calculate the levels of relative genes. The following are the sequence of primers: ZO-1 forward 5′-GCCGCTAAGAGCACAGCAA-3′, reverse 5′-GCCCTCCTTTTAACACATCAGA-3′, occludin forward 5′-ACAAGCGGTTTTATCCAGAGTC-3′, reverse 5′-GTCATCCACAGGCGAAGTTAAT-3′, RhoA forward 5′-AGCCTGTGGAAAGACATGCTT-3′, reverse 5′-TCAAACACTGTGGGCACATAC-3′, TNF-α forward 5′-GGAACACGTCGTGGGATAATG-3′, reverse 5′-GGCAGACTTTGGATGCTTCTT-3′, IL-1β forward 5′-AAGGGGACATTAGGCAGCAC-3′, reverse 5′-ATGAAAGACCTCAGTGCGGG-3′, IL-6 forward 5′- GGCGGATCGGATGTTGTGAT-3′, reverse 5′-GGACCCCAGACAATCGGTTG-3′, GAPDH forward 5′- AGGTCGGTGTGAACGGATTTG-3′, reverse 5′- GGGGTCGTTGATGGCAACA-3′.

### Western blot

Total proteins were extracted with RIPA lysis buffer (Solarbio) and quantified using a bicinchoninic acid (BCA) protein assay kit (Thermo Fisher Scientific Inc.). Subjected to 10% gel with sodium dodecyl sulfate polyacrylamide gel electrophoresis (SDS-PAGE), the proteins were transferred onto a polyvinylidene fluoride (PVDF) membrane. After inhibition with 5% nonfat milk, primary antibodies against ZO-1 (ab61357; 0.1–1 µg/ml; Abcam), occludin (ab242202; 1 µg/ml; Abcam), RhoA (ab54835; 1:100; Abcam), ROCK1 (21850-1-AP; 1:3000; Proteintech), ROCK2 (21645-1-AP; 1:6000; Proteintech), P-MLC2 (10906-1-AP; 1:2000; Proteintech), MLC2 (10906-1-AP; 1:2000; Proteintech), Bax (50599-2-Ig; 1:6000; Proteintech) and Bcl2 (26593-1-AP; 1:1500; Proteintech) were utilized to incubate the membranes at 4°C overnight. Thereafter, secondary antibodies were adopted to incubate the membranes on the next day. Finally, the protein bands were visualized using an enhanced chemiluminescence (ECL).

### Terminal-deoxynucleoitidyl Transferase Mediated Nick End Labeling (TUNEL) staining

The effects of oxycodone on apoptosis were assessed with TUNEL. In the beginning, bEND3 cells were immobilized and permeabilized with 4% paraformaldehyde and 0.1% citrate buffer (Sigma-Aldrich) containing additional 0.1% Triton X-100 (Sigma-Aldrich), respectively. Thereafter, the cells were rinsed by phosphate buffer saline (PBS) and incubated in TUNEL reaction solution for 1 h. With the adoption of an inverted fluorescence microscope, the images of positive apoptotic cells were photographed.

### Statistical analysis

Data collected from experiments were presented as mean ± SD and analyzed with GraphPad Prism 8.0 software (GraphPad software, Inc.). The comparisons among different group were performed by one-way ANOVA and Tukey’s test. Differences with a value of p < 0.05 were viewed to show statistical significance.

## Results

### Oxycodone attenuated cell activity damage in OGD/R-induced brain microvascular endothelial cells

**Figure 1.**
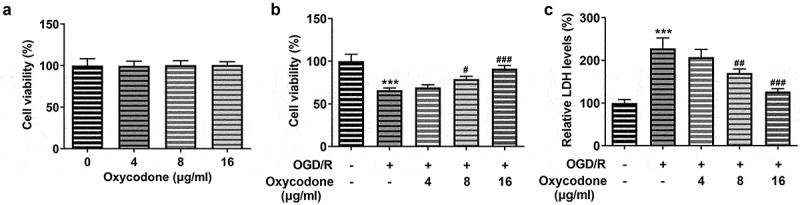
Oxycodone attenuated cell activity damage in OGD/R-induced brain microvascular endothelial cells. (a) The viability of bEND3 cells was detected using MTT. (b) The viability of OGD/R-induced bEND3 cells was detected by using MTT. (c) The relative LDH level was detected using LDH assay. ***P < 0.05 vs. control; #P < 0.05, ##P < 0.01 and ###P < 0.001 vs. OGD/R.

For the reliability of results in the following experiments, the viability of bEND3 cells was firstly detected by using CCK-8 and LDH assay. As Figure 1(a) demonstrated, there was no significant difference in bEND3 cell viability after treatment with 4–16 μg/ml oxycodone. Under OGD/R conditions, cell viability was significantly decreased compared to the control (Figure 1(b)). However, treatment with oxycodone increased cell viability that was reduced by the OGD/R condition. It is noted that oxycodone exhibited promotive effects on OGD/R-induced bEND3 cells in a concentration-dependent manner. Moreover, the increased LDH level caused by OGD/R was subsequently reduced by oxycodone treatment. Notably, oxycodone with a concentration of 16 μg/ml contributed to the lowest level of LDH in comparison with other groups (Figure 1(c)).

### Oxycodone improved the permeability damage of OGD/R-induced brain microvascular endothelial cells

**Figure 2.**
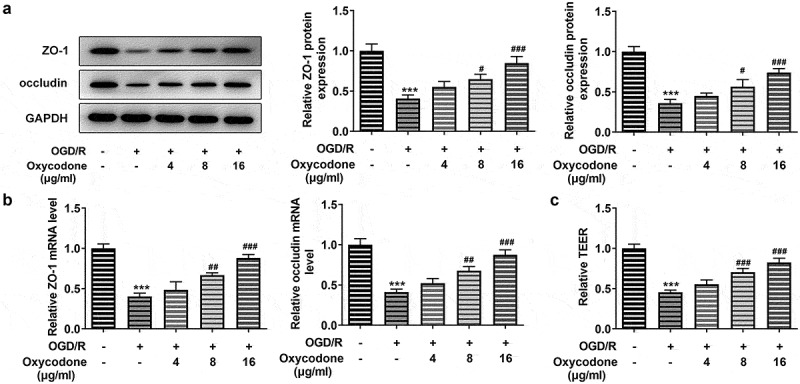
Oxycodone improved the permeability damage of OGD/R-induced brain microvascular endothelial cells. The expressions of ZO-1 and occludin were measured using Western blot (a) and RT-qPCR (b). (c) The relative TEER was detected using TEER analysis. ***P < 0.05 vs. control; #P < 0.05, ##P < 0.01 and ###P < 0.001 vs. OGD/R.

With the application of RT-qPCR and Western blot, the mRNA and protein expressions of tight junction proteins, including ZO-1 and occludin were detected. Compared with control group, the mRNA and protein expression levels of ZO-1 and occludin gained a huge growth in bEND3 cells after OGD/R induction (Figure 2(a,b)). Nevertheless, the upregulated expressions of ZO-1 and occludin caused by OGD/R induction were then diminished by oxycodone treatment. What’s more, OGD/R contributed to serious permeability damage, while oxycodone treatment improved that (Figure 2(c)). The above-mentioned results suggested that oxycodone could alleviate the permeability damage of OGD/R-induced brain microvascular endothelial cells.

### Oxycodone reduced inflammatory cytokine release and apoptosis of OGD/R-induced brain microvascular endothelial cells

**Figure 3.**
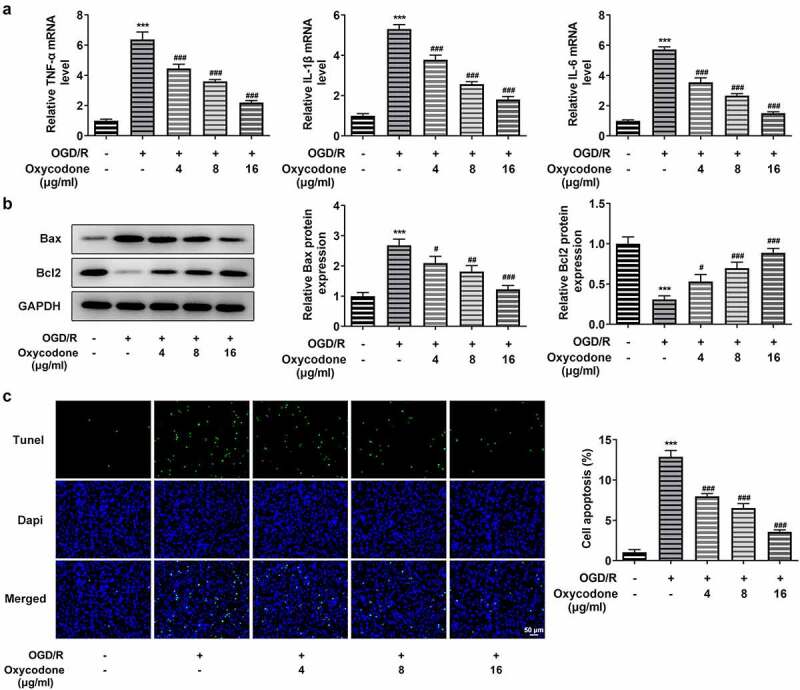
Oxycodone reduced inflammatory cytokine release and apoptosis of OGD/R-induced brain microvascular endothelial cells. (a) The levels of TNF-α, IL-1β and IL-6 were detected using RT-qPCR. (b) The expressions of apoptosis-related proteins were detected using Western blot. (c) The apoptosis was detected using TUNEL. ***P < 0.05 vs. control; #P < 0.05, ##P < 0.01 and ###P < 0.001 vs. OGD/R.

The inflammatory cytokine levels including TNF-α, IL-1β and IL-6 were assessed with RT-qPCR. Apoptosis was measured by Western blot and TUNEL staining. It is worth noting that inflammatory factor expression was significantly increased in OGD/R-induced cells in comparison to the control group, whereas oxycodone-treated bEND3 cells showed a dose-dependent decrease in inflammatory factor expression compared to the OGD/R-induced group (Figure 3(a)). As Figure 3(b) demonstrated, OGD/R induction upregulated Bax expression but downregulated Bcl2 expression, which were then reversed by oxycodone treatment. Moreover, the apoptosis in OGD/R-induced bEND3 cells was inhibited by oxycodone treatment in dose-dependent manner (Figure 3(c)).

### Oxycodone decreased the RhoA/ROCK/MLC2 signal in OGD/R-induced brain microvascular endothelial cells

**Figure 4.**
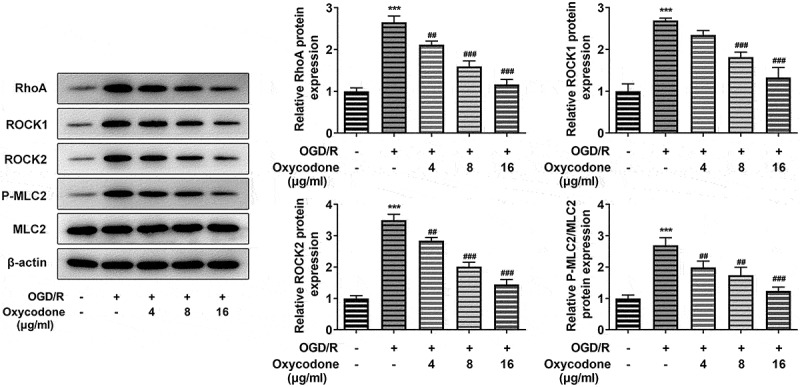
Oxycodone decreased the expressions of RhoA/ROCK/MLC2 in OGD/R-induced brain microvascular endothelial cells. The expressions of RhoA, ROCK1, ROCK2, P-MLC2 and MLC2 were measured using Western blot. ***P < 0.05 vs. control; ##P < 0.01 and ###P < 0.001 vs. OGD/R.

In order to investigate the effects of oxycodone on RhoA/ROCK/MLC2 signal in OGD/R-induced bEND3 cells, the protein expression levels of RhoA, ROCK1, ROCK2, P-MLC2 and MLC2 were measured by using Western blot. Compared with control group, OGD/R induction greatly enhanced the expression levels of RhoA, ROCK1, ROCK2 and P-MLC2/MLC2, while these increased expressions were then gradually decreased with the change of oxycodone concentration (Figure 4).

### Oxycodone alleviated cell activity damage in OGD/R-induced brain microvascular endothelial cells through RhoA/ROCK/MLC2 signal

**Figure 5.**
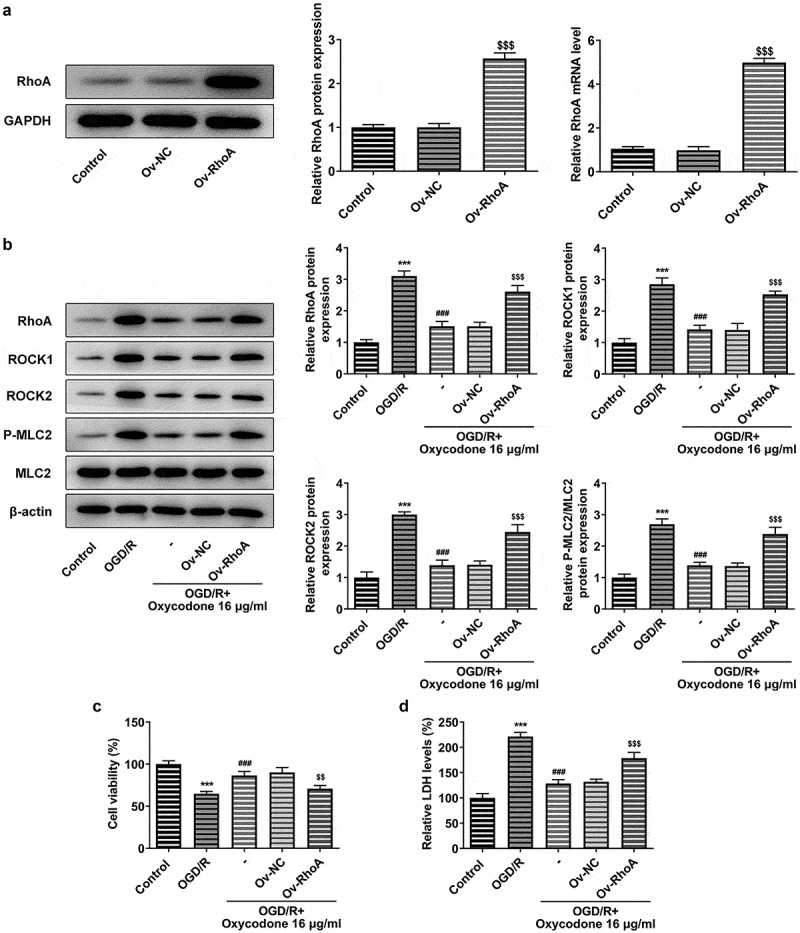
Oxycodone alleviated the damage of activity and toxicity in OGD/R-induced brain microvascular endothelial cells through RhoA/ROCK/MLC2 signal. (a) The mRNA and protein expressions of RhoA were detected using RT-qPCR and Western blot, respectively. (b) The protein expressions of RhoA, ROCK1, ROCK2, P-MLC2 and MLC2 were detected using Western blot. (c) The viability was detected using MTT. (d) The relative LDH level was detected using LDH assay. ***P < 0.05 vs. control; ###P < 0.001 vs. OGD/R; ^$$^P < 0.01 and ^$$$^P < 0.001 vs. Ov-NC.

Overexpression plasmids specific to RhoA were used to transfect bEND3 cells. Obviously, the RhoA mRNA and protein expression levels gained a huge growth after transfection of the overexpressed RhoA plasmid into bEND3 cells in comparison with Ov-NC (Figure 5(a)). According to Figure 5(b), oxycodone could inhibit RhoA, ROCK1, ROCK2 and P-MLC2/MLC2 expression levels in OGD/R-induced bEND3 cells. However, RhoA overexpression were then partially abolished the suppressive effects of oxycodone, evidenced by the enhanced expressions of RhoA, ROCK1, ROCK2 and P-MLC2/MLC2 in OGD/R+ oxycodone +Ov-RhoA. Likewise, the cell viability in OGD/R+ oxycodone group was then inhibited after transfection with RhoA overexpression plasmids (Figure 5(c)). Furthermore, the relative LDH level was significantly enhanced by OGD/R induction. In comparison with OGD/R+ oxycodone +Ov-NC, the LDH level caused by oxycodone treatment was subsequently reversed in Ov-RhoA cells (Figure 5(d)).

### Oxycodone alleviated the permeability damage in OGD/R-induced brain microvascular endothelial cells via RhoA/ROCK/MLC2 signal

**Figure 6.**
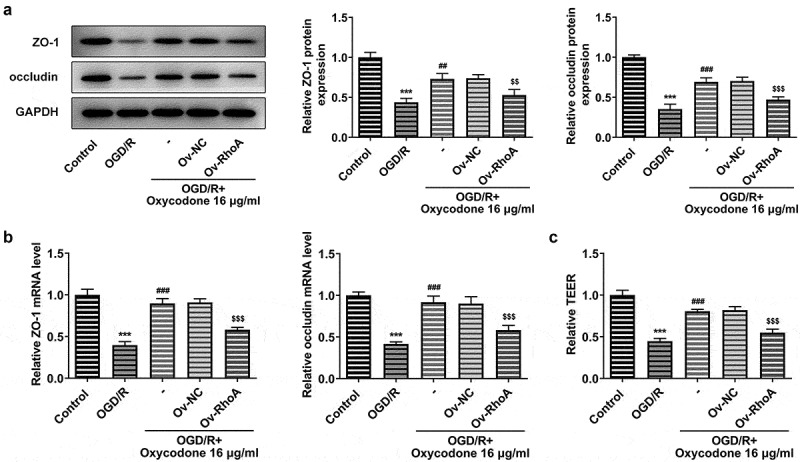
Oxycodone alleviated the permeability damage in OGD/R-induced brain microvascular endothelial cells through RhoA/ROCK/MLC2 signal. The expressions of ZO-1 and occludin were measured using Western blot (a) and RT-qPCR (b). (c) The relative TEER was detected using TEER analysis. ***P < 0.05 vs. control; ##P < 0.01 and ###P < 0.001 vs. OGD/R; ^$$^P < 0.01 and ^$$$^P < 0.001 vs. Ov-NC.

The permeability-associated protein ZO-1 and occludin expression were detected by Western blot and RT-qPCR after transfection of the overexpression RhoA plasmid into bEND3 cells. Compared with OGD/R group, the ZO-1 and occludin expressions gained a huge growth after oxycodone treatment. Nevertheless, these protein expressions in OGD/R+ oxycodone +Ov-RhoA group were downregulated by RhoA overexpression compared with OGD/R+ oxycodone +Ov-NC group, revealing that RhoA overexpression reversed the promotive effects of oxycodone on tight junction proteins (Figure 6(a,b)). Besides, RhoA overexpression also decreased the relative TEER level in OGD/R+ oxycodone +Ov-NC (Figure 6(c)).

### Oxycodone reduced inflammatory cytokine release and apoptosis in OGD/R-induced brain microvascular endothelial cells through RhoA/ROCK/MLC2 signal

**Figure 7.**
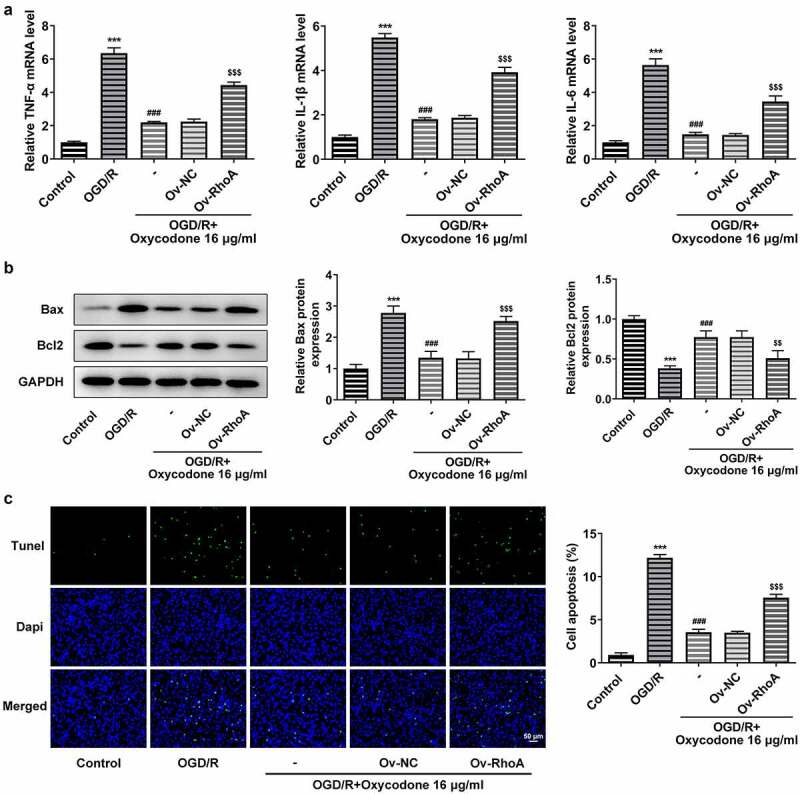
Oxycodone reduced inflammatory cytokine release and apoptosis in OGD/R-induced brain microvascular endothelial cells via RhoA/ROCK/MLC2 signal. (a) The levels of TNF-α, IL-1β and IL-6 were detected using RT-qPCR. (b) The expressions of apoptosis-related proteins were detected using Western blot. (c) The apoptosis was detected using TUNEL. ***P < 0.05 vs. control; ###P < 0.001 vs. OGD/R; ^$$^P < 0.01 and ^$$$^P < 0.001 vs. Ov-NC.

The study replicated previous experiments to verify that RhoA overexpression inhibited the effect of oxycodone, demonstrating that oxycodone worked through RhoA signaling. As Figure 7(a) shown, oxycodone exhibited inhibitory effects on the high levels of TNF-α, IL-1β and IL-6 caused by OGD/R induction. However, RhoA overexpression reversed the inhibitory effects of oxycodone on inflammatory response in OGD/R+ oxycodone +Ov-RhoA group. In addition, oxycodone treatment downregulated Bax in OGD/R-induced bEND3 cells but upregulated Bcl2 expression, which was then reversed after transfection with RhoA overexpression plasmids (Figure 7(b)). Moreover, RhoA overexpression also increased the cell apoptosis in OGD/R-induced bEND3 cells with oxycodone treatment in contrast to OGD/R+ oxycodone +Ov-NC (Figure 7(c)).

## Discussion

This study was the first to investigate the effects of oxycodone via RhoA/ROCK/MLC2 on OGD/R-induced permeability damage and apoptosis in brain microvascular endothelial cells. The study measured cell viability, apoptosis, permeability and pathway-related protein expression, respectively. The results indicated that oxycodone significantly inhibited OGD/R-induced permeability damage and inflammatory response in bEND3 cells, and that this effect was achieved by inhibiting RhoA/ROCK/MLC2 signaling.

Oxycodone is a pure semi-synthetic opioid agonist derived from thebaine with affinity for the kappa receptor, and to a lesser degree for the mu receptor [25]. Oxycodone could be utilized for the management of patients with pain and opioid-induced bowel dysfunction [26]. And the study found that oxycodone could reduce inflammatory damage [27]. In this study, we firstly adopted oxycodone to treat bEND3 cells and OGD/R-induced bEND3 cells, finding that oxycodone had no influence on bEND3 cells but dose-dependently protected OGD/R-induced bEND3 cell viability. The expression levels of inflammatory factors TNF-α, IL-1β and IL-6 were significantly increased in brain microvascular epithelial cells after OGD/ R induction. In contrast, when the concentration of oxycodone increased, the expression levels of inflammatory factors subsequently decreased. This suggested that oxycodone had a significant inhibitory effect on the OGD/R-induced inflammatory response in brain microvascular epithelial cells. In addition, oxycodone could upregulate the expression levels of tight junction proteins and reduce pulmonary microvascular permeability [17]. Similarly, the study revealed a significant decrease in ZO-1 and occludin expression levels after OGD/R induction, and in contrast, a dose-dependent increase in ZO-1 and occludin expression levels after oxycodone treatment. This suggested that oxycodone could effectively improve brain microvascular endothelial cell permeability.

To further investigate the mechanism of oxycodone action on brain microvascular endothelial cells, studies were conducted to further reverse validate the RhoA/ROCK/MLC2 signaling pathway. RhoA/ROCK signaling pathway has close relation with a variety of cell functions, including actin cytoskeleton recombination, movement and morphology [28]. It was discovered that Y-27632, a specific inhibitor of RhoA signaling pathway, has been proved to alleviate in vitro cerebral barrier dysfunction during ischemic injury [29]. Additionally, a study confirmed that the inhibition of Rho/ROCK signaling pathway might be an effective method for the treatment of OGD-induced brain microvascular endothelial cells [30]. Notably, previous studies have investigated the effects of RhoA/ROCK/MLC2 in blood-brain barrier injury after cerebral ischemia/reperfusion [21–23]. More importantly, available report suggested that oxycodone ameliorated myocardial ischemia-reperfusion injury in rats via RhoA/ROCK1 signaling [20]. In the present study, we discovered that oxycodone downregulated the expression levels of RhoA/ROCK/MLC2 in OGD/R-induced brain microvascular endothelial cells, demonstrating for the first time that oxycodone could affect RhoA/ROCK/MLC2 signaling in brain microvascular endothelial cells. Moreover, the original effect of oxycodone on OGD/R-induced brain microvascular endothelial cell permeability and apoptosis was significantly reversed upon activation of RhoA signaling, further demonstrating that oxycodone improved OGD/R-induced brain microvascular endothelial cell permeability damage and apoptosis through inhibition of RhoA expression.

## Conclusion

After a series of studies, it could be tentatively concluded that oxycodone significantly ameliorated OGD/R-induced permeability damage and apoptosis in brain microvascular endothelial cells through inhibition of RhoA/ROCK/MLC2 signal. Although this study was conducted primarily in an OGD/R-induced brain microvascular endothelial cell model, it was undeniable that the first study of this mechanism of oxycodone provided an idea for the treatment of cerebral ischemia-reperfusion diseases. In the meantime, in future studies, investigators will perform more in-depth studies on this mechanism in the animal model.

## Highlights

- Oxycodone relieves OGD/R-induced permeability damage in bEND3 cells.
- Oxycodone reduces OGD/R-induced apoptosis in bEND3 cells.
- Oxycodone inhibits Rhoa/ROCK/MLC2 expression in OGD/R-induced bEND3 cells.
